# Beyond the reducing valve: towards a computational neurophenomenology of altered states via deep neural networks

**DOI:** 10.3389/fpsyg.2026.1819038

**Published:** 2026-05-20

**Authors:** Keisuke Suzuki

**Affiliations:** Center for Human Nature, Artificial Intelligence and Neuroscience (CHAIN), Hokkaido University, Sapporo, Japan

**Keywords:** altered states of consciousness, computational neurophenomenology, deep convolutional neural networks (DCNNS), ego dissolution, generative adversarial networks (GAN), hallucinations, perceptual reality monitoring, reducing valve

## Abstract

Altered states of consciousness, including hallucinations, psychedelic experiences, and ego dissolution, differ qualitatively, yet no unified computational framework describes what varies and along which dimensions. Computational phenomenology (CP) has emerged as a promising bridge between first-person experience and computational models, yet current formalisations rely predominantly on the free energy principle (FEP). This paper proposes the C × G × D framework, drawing on three functional roles in deep neural networks: a Classifier (C) that extracts features from sensory input, a Generator (G) that synthesises internal representations, and a Discriminator (D) that judges whether a representation originates externally or internally. Phenomenological differences across altered states are redescribed as variations in the objective functions, constraints, and thresholds of these components. The framework reformulates Huxley’s ‘reducing valve’ metaphor: relaxation of C’s constraint exposes normally hidden ‘effective causes,’ producing psychedelic geometric patterns; G’s prior governs hallucinatory veridicality; and D instantiates Perceptual Reality Monitoring. Three hallucination mechanisms—psychedelic, neurodegenerative, and schizophrenia-type—are predicted from distinct parameter configurations. Testable hypotheses derived from iterative-optimisation psychophysics and an extension to ego dissolution are presented. By foregrounding the plurality of objective functions and architectures, the C × G × D framework complements FEP-centred CP and provides a scaffold for translating phenomenology into experimentally manipulable variables.

## 1 Introduction

More than 30 years after the identification of Neural Correlates of Consciousness (Crick and Koch, 1990), no formal translation rules have been established between the qualitative content experienced by subjects and the variables described by computational or neural models. The explanatory gap (Levine, 1983), also known as the hard problem of consciousness (Chalmers, 1995), is, at its methodological core, a problem of translation: bridging first-person and third-person descriptions. Explanatory theories such as Integrated Information Theory, Global Neuronal Workspace, higher-order theories, and predictive processing / the Free Energy Principle (FEP; Friston, 2005, 2010) have each proposed mechanisms for consciousness (Seth and Bayne, 2022), yet they do not provide an ‘operational grammar’ that maps specific qualitative differences onto manipulable model parameters. Here, altered states of consciousness, and visual hallucinations in particular, serve as an ideal touchstone. Hallucinations are not monolithic: psychedelics induce kaleidoscopic geometric patterns, while patients with Parkinson’s disease (PD) or dementia with Lewy bodies (DLB) report visual hallucinations of people or animals indistinguishable from reality, qualitatively distinct experiences subsumed under the single label ‘hallucination.’ If this diversity is not accidental, but arises from specific parameter variations in the perceptual generative process, an operational framework that describes ‘what varies and along which dimensions’ (for instance, why psychedelic experience is geometric, whereas Parkinson’s-disease hallucinations are veridical) is precisely what is called for.

In response to this methodological impasse, the programme of ‘naturalising phenomenology’, connecting phenomenological inquiry with the methods of empirical science, gained momentum. As a pioneering contribution to this programme, Varela proposed neurophenomenology, in which first-person phenomenology and third-person neuroscience mutually constrain one another (Varela, 1996). Since then, a suite of methods aimed at improving the precision and reproducibility of subjective reports has been developed: phenomenological cluster analysis (Lutz, 2002), front-loaded phenomenology (Gallagher, 2003), and micro-phenomenological interviewing (Petitmengin, 2006), among others. Nonetheless, a decisive means of supplying formal, functional ‘translation rules’ between high-resolution phenomenological description and the dynamic description of complex neural networks remains lacking. Verbal, qualitative descriptions do not map straightforwardly onto descriptions of neural activity, and an intermediate representation capable of shuttling between the two has long been sought.

Building on this accumulated methodology, computational phenomenology (CP) has recently emerged as a promising candidate for such a bridge. CP embeds the structure of experience (constitution) within computational models and translates subjective differences into the architectures and objective functions of those models, thereby formalising the ‘epistemological dialogue’ between phenomenology and neuroscience (Ramstead et al., 2022). This direction can be situated as an attempt to implement Varela’s ideal of mutual constraint on the concrete medium of deep-learning models. Unlike approaches that reduce everything to a single principle such as FEP, CP opens model selection itself as a tool for phenomenological description (Beckmann et al., 2023; Sandved-Smith et al., 2025).

The present paper positions computational phenomenology as the intermediate representation introduced above, and contends that deep-learning models, in particular deep convolutional neural networks (DCNNs)—multi-layered artificial neural networks that process images through successive layers of learned spatial filters —, can serve as implementable substrates for ‘generative passages’: methodological circuits in which the structures of first-person experience and the formal structures of computational models reciprocally constrain one another (Ramstead et al., 2022). DCNNs, although designed for engineering purposes, have been shown to form representational spaces that are remarkably congruent with the hierarchical representations of the biological visual system (Yamins and DiCarlo, 2016; Kriegeskorte, 2015; Khaligh-Razavi and Kriegeskorte, 2014). What matters here is that these models are not mere classifiers: they can be instantiated as generative processes encompassing optimisation procedures, constraints, and priors. This opens a path towards explaining differences in subjective experience not as correlations, but as constructions.

The present paper uses visual hallucinations as a concrete testbed for this programme. DeepDream / Activation Maximisation applied to DCNNs has been integrated with virtual reality (VR) to construct a Hallucination Machine that selectively presents hallucinatory visual distortions without pharmacological intervention, and subjective reports have indicated qualitative proximity to psychedelic experience (Suzuki et al., 2017). Models coupling DCNNs with deep generator networks have been used to operationally reproduce hallucinations of different aetiologies (neurodegenerative, visual-impairment, psychedelic) along the dimensions of veridicality, spontaneity, and complexity (Suzuki et al., 2024). These studies suggest that qualitative differences among hallucinations can be mapped onto computational variables such as network architecture, optimisation procedure, and iteration conditions. (These architectures and algorithms are defined in Sections 3 and 4.)

At the same time, FEP, on which mainstream computational phenomenology currently relies, is a powerful unifying framework, yet criticisms have also been raised regarding its epistemological status (Andrews, 2021; Bruineberg et al., 2021), and the blind spots introduced by convergence on a single principle cannot be overlooked. Accordingly, this paper does not reject FEP but instead adopts the strategy of treating the architecture of deep learning itself as a ‘phenomenological descriptor.’ Specifically, taking three computational roles in deep learning—classification, generation, and discrimination—as manipulable axes, it constructs the C × G × D framework, which describes qualitative differences among altered states as variations in objective functions, constraints, and thresholds.

This paper contributes beyond existing work in three ways. Computational phenomenology as such is attributed to Ramstead et al. (2022); the idea of the generative adversarial network (GAN) as a reality monitor to Gershman (2019); and hallucination simulation via DCNN visualisation to Suzuki et al. (2017, 2024). First, it integrates these separate findings into a three-way framework (Classifier C × Generator G × Discriminator D) and presents a formulation in which phenomenological differences are described as parameter variations within a single architectural schema. Second, it concretely designs an iterated-convergence psychophysical experiment using each variable of the C × G × D framework (degree of classificatory-feature exposure, strength of the natural image prior, adoption threshold) as an independent variable, thereby securing the framework’s falsifiability. Third, it foregrounds the ‘plurality of objective functions and architectures’ that FEP-centrism tends to overlook, repositioning computational phenomenology not as a reduction to a single principle but as an exploration of design space. These integrations, experimental designs, and theoretical reorientations are not contained in the individual prior studies and constitute the distinctive contribution of this paper.

The paper is organised as follows. Section 2 surveys the main currents of computational phenomenology and identifies the strengths and limitations of FEP-centrism. Section 3 reviews constructive research on visual hallucinations and charts the methodological innovations that coupling DCNNs with deep generator networks has brought to hallucination research. Section 4 offers an integrated discussion of the C × G × D framework: its epistemological premises (section 4.1), Classifier C and the reducing-valve metaphor (section 4.2), the veridicality constraint imposed by Generator G and the C–G asymmetry (section 4.3), Discriminator D and Perceptual Reality Monitoring (section 4.4), and connections with existing theories of neural computation (section 4.5). Section 5 develops psychophysical experimental designs and testable hypotheses derived from the three-axis operational space (section 5.1), extends the reducing-valve reformulation to ego dissolution (section 5.2), and discusses limitations and future directions (section 5.3). Section 6 presents the conclusions.

## 2 Computational phenomenology: a critical review

Computational phenomenology is a research programme that goes beyond externalising first-person phenomenological descriptions as ‘data to be explained’: it seeks to embed the structure (constitution) of experience within computational models and thereby to construct intermediary terms that can shuttle between third-person (behavioural, neural, environmental) and first-person perspectives. This enterprise is best understood as pushing Varela’s neurophenomenological ideal of ‘mutual constraints’ in a constructive, formal direction (Varela, 1996). At least two technical and theoretical preconditions have underpinned the recent rise of CP. The first is the maturation of theories that cast perception and cognition in the vocabulary of statistical machine learning, exemplified by FEP and predictive processing. These frameworks have opened the way to describing structural features of subjective experience (prior, precision, prediction error) as manipulable parameters of generative models. The second is the rapid advance of deep-learning technology, which has brought artificially generated images, videos, and sounds to a quality directly comparable with human perceptual experience, that is, a quality amenable to phenomenological verification. With both conditions in place, CP has become feasible not merely as a theoretical framework but as a constructive practice: a cycle in which computational models reconstruct the structure of experience and first-person reports serve as the test.

### 2.1 FEP-centred formalisation

The most influential current within CP at present is generative modelling grounded in FEP and active inference (the corollary of FEP whereby organisms act on the world to fulfil their predictions). CP is positioned as methodological naturalisation, harnessing phenomenological description as a constraint on model building and thereby rendering it continuous with empirical science (Ramstead et al., 2022). Concretely, Ramstead et al. (2022) formalise the ‘constitution’ of experience, a concept traditionally handled by phenomenology, as inference within a generative model, demonstrating that the inferential and interpretive processes required for a given type of experience to obtain can be constructed as a computational model. The term ‘generative passages’ refers to a reciprocal circuit in which (i) first-person descriptions specify the model’s structure, latent variables, and constraints, (ii) the model’s inferential dynamics yield predictions about the temporal structure of experience, and (iii) those predictions are falsifiable by behavioural, physiological, and neural data. The strength of this framework is that it treats subjective reports not merely as supplementary data in a correlation search but as a formal constraint on model identification.

As a more principled extension of these ‘generative passages,’ a programme for quantifying phenomenological differences has been proposed that treats them as beliefs (approximate posteriors) of a generative model and measures the distances between them with the tools of information geometry (da Costa et al., 2024). The crux of their argument is that even without changing experiential content (the mean), a shift in confidence (precision) alone can substantially alter the distance between beliefs. This opens a mathematically tractable avenue for addressing phenomenologically significant differences (presence, confidence, the onset of error) that are determined not by content alone but also by precision. In the longer term, they also sketch a programme in which a subject’s phenomenology is characterised as a region in ‘phenomenological space’ and deviant experiences (delusions, aberrant percepts) are described as distances from the typical region. This direction has been pushed further in ‘deep computational neurophenomenology,’ in which phenomenological data, physiological data, and computational models circulate within a single analytic loop (Sandved-Smith et al., 2025). By formalising advanced meditative states of consciousness through an active-inference model, this work demonstrates that FEP-centred CP can be applied to the description of specific states of consciousness, going beyond a purely theoretical construct.

### 2.2 Non-representationalist perspectives and FEP critiques

FEP-centred CP formalises experience as ‘the structure of inference,’ but this formalisation harbours several philosophical tensions. First, the Kantian concept of ‘constitution’ (Konstitution) extensively used in Ramstead et al.’s framework indicates that the predictive-processing / FEP framework implicitly contains a neo-Kantian epistemological structure (Zahavi, 2018; cf. Swanson, 2016), one rooted in a philosophical tradition different from the Husserlian phenomenology on which Varela drew, a stance that foregrounds intentionality and embodiment, treating experience as a negotiation between the lived body and the world. Second, Andrews (2021) questions the epistemological status of FEP itself, arguing that FEP should be understood not as a substantive empirical theory but as a model structure (mathematical formalism); similarly, Bruineberg et al. (2021) demonstrate that the Markov blanket formalism (the statistical boundary separating internal from external states) central to FEP conflates distinct mathematical and metaphysical senses; on this view, FEP is not the sole principle that ‘explains’ consciousness but merely one among many possible modelling formalisms. This epistemological critique resonates with the tension identified by Di Paolo et al. (2022) between FEP’s convergence on steady states and the historicity and open-ended transformation central to enactivism. These critiques intimate that two methodological premises (‘experience as inference’ and ‘experience as enactment through the body’) can compete within CP. Against this inference-centred formalisation, the line of work described below explores the possibility of a computational phenomenology that treats experience as ‘the reactivation of bodily habits’ and does not pass through representationalism.

An alternative proposal reserves judgement on convergence towards FEP-centred formalisation and repositions deep learning as an independent exploratory source for experience research (Beckmann et al., 2023). Beckmann et al. (2023) distinguish three ‘sources of inquiry’ (first-person phenomenology, third-person neurophysiology, and computational models) and propose constructing CP as an exclusive dialogue between phenomenology and computation (especially deep learning), provisionally bracketing the neurophysiological source. Their aim is to reject the cognitivist and neuro-representationalist view of deep learning as an encoding device for external-world representations and to reinterpret the learning and inference of networks as processes through which ‘habits’ sedimented in the lifeworld are reactivated. Furthermore, they positively evaluate the opacity (black-box character) of artificial neural networks not as a defect but as parallelling the phenomenological character of pre-reflective, non-decomposable cognitive processes, and propose a methodology that uses deep-learning interpretation techniques to explore correspondences with specific experiential structures. This line of work is congenial to the constructive approach taken in this paper, insofar as it directly connects the descriptive precision of phenomenology with the constructive power of deep learning, rather than unifying CP into a general theory of inference.

### 2.3 Objective functions × Architectures: the position of this paper

The explanatory power of the FEP-centred approach (notably, precision weighting for the modulation of attention and sensory gain, and nested optimisation for multi-timescale integration) represents a significant advance. At the same time, that very unity carries limitations. Convergence on a single objective function makes it difficult to distinguish, at the algorithmic level, the phenomenological differences wrought by different architectures and objective functions. An important corrective is provided by the research programme that treats deep neural networks as a language for expressing computational theories of the brain, systematically manipulating four design dimensions (objective function, architecture, learning rule, and training data) and testing the resulting models against neural and behavioural data (Kriegeskorte, 2015; Richards et al., 2019; Doerig et al., 2023). A large-scale study that systematically compared diverse models has empirically demonstrated that qualitatively different architectures and objective functions can predict the brain’s visual representations to a nearly equivalent degree, confirming that the design space is genuinely multidimensional (Conwell et al., 2024). Accordingly, it is methodologically more precise to situate FEP not as ‘the brain’s sole principle’ but as one class (a powerful class, to be sure) within the broad design space of objective functions × architectures × learning rules. This repositioning liberates CP from ‘reduction to a single principle’ and renders model selection for explaining differences in experience more explicit and testable.

In light of the above, this paper connects FEP-centred formalisation and non-representationalist CP not as mutually exclusive opposites but on the premise of plurality in objective functions and architectures. The crux is to treat not the single objective of ‘free-energy minimisation’ as the repository of phenomenological difference, but rather the (i) objective function (what is being optimised) and (ii) architecture (through what kind of recurrence and interaction inference/generation proceeds) of deep-learning models as manipulable phenomenological descriptors. Section 4 develops this stance in detail.

## 3 Artificial neurophenomenology in practice

As argued in Section 2, computational phenomenology can be redefined as a research programme that translates phenomenological differences into manipulable variables by explicitly selecting and substituting pairs of objective functions and architectures, rather than subsuming experience under a single objective function. This section deploys that framework constructively, taking visual hallucinations as a case study, and manipulates (i) the representational hierarchy, (ii) the generative constraint (prior), and (iii) the dynamics of iterative optimisation within deep-learning models to reconstruct changes in the quality of experience (veridicality, spontaneity, complexity). In other words, hallucinations are not treated as mere noise or by-products of pathology; instead, they are situated as a point within the ‘space of perceptual possibilities’ that becomes manifest when design variables of the generative process are varied, thereby furnishing a scaffold that connects first-person description to the formal structure of the model.

### 3.1 VR × DeepDream as a hallucination machine

**Figure 1:**
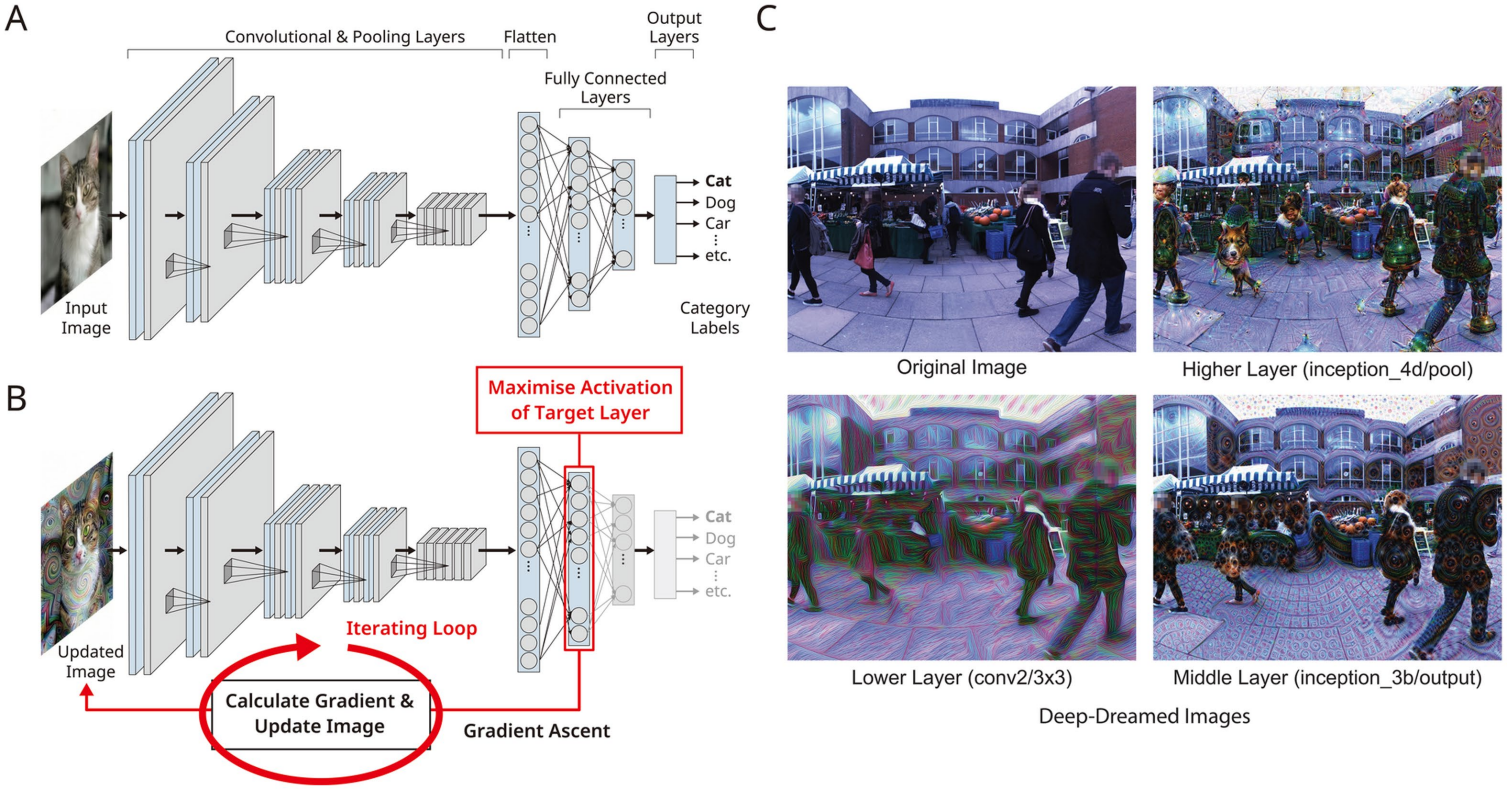
Deep convolutional neural network (DCNN) and the DeepDream algorithm. **(A)** Architecture of the deep convolutional neural network. **(B)** Schematic illustration of the DeepDream algorithm. **(C)** Examples of images generated by the DeepDream algorithm, adapted from Suzuki et al., 2017. By selecting different target layers, the generated images contain higher- or lower-level visual features learnt in the DCNN.

An early and representative example is the Hallucination Machine, which integrates VR video with DeepDream-family algorithms (Figure 1; Suzuki et al., 2017). The system amplifies internal representations (features) in a deep convolutional neural network via gradient ascent (an iterative optimisation that adjusts the input to maximise a target activation), generating visual alterations in which specific patterns ‘intrude’ upon the input image. This operation is a form of feature visualisation based on Activation Maximisation (AM), first proposed by Erhan et al. (2009) and subsequently popularised under the rubric of DeepDream (Mordvintsev et al., 2015). What is crucial is that the hallucinatory patterns produced here are not designed by humans in the manner of conventional altered-state simulators; rather, they emerge from a neural network trained to accomplish the specific task of object recognition. That is, internal features acquired through the learning process, representations beyond human design intent, are projected onto the input image, giving rise to unpredictable and alien visual alterations. It is this ‘emergent’ character that endows the output with a computational significance fundamentally different from mere filter processing or artistic effects (details in section 4.2). This output was presented immersively as a 360-degree panoramic video via a head-mounted display, drawing on the design of the Substitutional Reality system (Suzuki et al., 2012). The updating of the visual field in response to head movements (visuo-motor coupling) generated a sense of presence (Froese et al., 2012), enabling computationally controlled visual alterations to arise for participants as ‘experienced hallucinations.’

Although its output takes the form of pixel images, the claim is not that conscious experience arises at the level of early visual cortex or the retina. Rather, given that hallucinatory experience takes a ‘perception-like’ form and that neural substrates are partially shared between imagination and perception (Dijkstra et al., 2022), it is highly probable that top-down processing spanning the visual hierarchy is involved in the generation of hallucinations. The fact that the basic form constants of psychedelic hallucinations (Klüver, 1926) can be mathematically derived from the neural architecture of V1 (Bressloff et al., 2001) provides evidence for the neural substrate of this hierarchical processing. What the Hallucination Machine manipulates are the computational correlates of such hierarchical processing; it makes no commitment regarding the final site at which experience is generated.

The significance of this VR implementation lies in its ability to reproduce psychedelic visual alterations as an external stimulus through computational manipulation, without pharmacological intervention. By isolating the hallucinatory experience as the product of a specific computational operation rather than as a multivariable pharmacological perturbation, the phenomenology of hallucinations can be directly linked to computational descriptions of the form: ‘at which representational level, what is being optimised, and to what degree of iteration does convergence occur’ (Suzuki et al., 2017). Leveraging this methodological advantage, independent replications have confirmed elevated brain-activity entropy and modulation of functional connectivity networks during the viewing of DeepDream-processed video, demonstrating proximity to the psychedelic state at the neurophysiological level (Greco et al., 2021). The prospect of applying AI-generated virtual psychedelic experiences to clinical use has been outlined (Riva et al., 2026).

### 3.2 Modelling aetiologically distinct hallucinations

**Figure 2:**
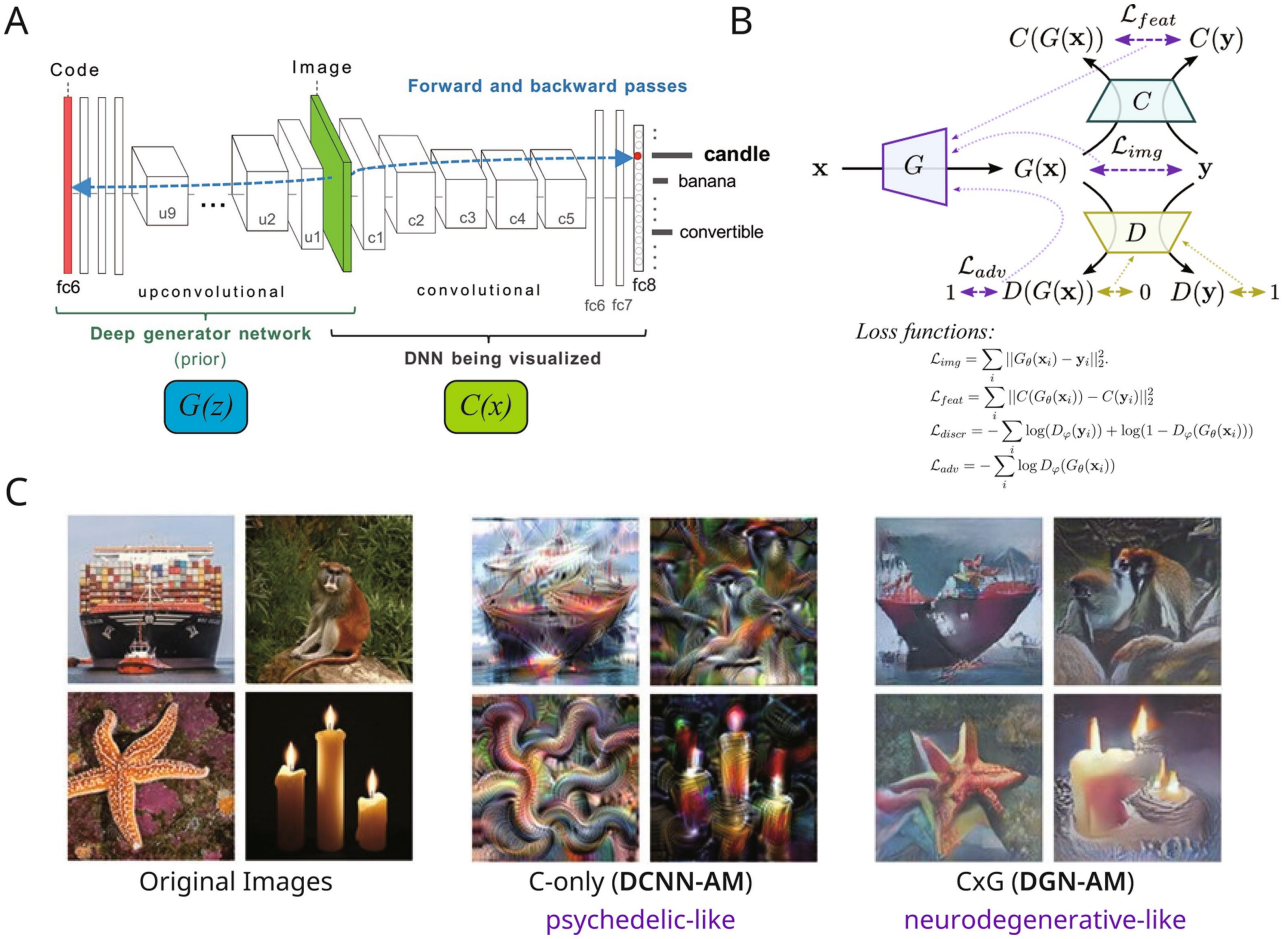
The DGN-AM architecture, C × G × D training schema, and comparison of generated images. **(A)** The DGN-AM architecture, adapted with permission from Nguyen et al. (2016), with classifier C and generator G indicated. **(B)** Training schema of the C × G × D architecture, adapted with the permission from from Dosovitskiy and Brox, 2016, with loss function equitation added below. G learns to synthesise images similar to the training data, while D learns to distinguish G’s outputs from real images. D is used during training to shape G’s generative prior; at inference time **(A)**, C and G interact directly. **(C)** Comparison of images generated by C-only activation maximisation (DCNN-AM) and C × G activation maximisation (DGN-AM). C-only (DCNN-AM) produces non-photorealistic, pattern-like images reflecting lower- and higher-level visual features (psychedelic-like), while C × G (DGN-AM) produces photorealistic images constrained by the generator’s natural image prior (neurodegenerative-like) (adapted from Suzuki et al., 2024, Supplementary materials).

DeepDream-type distortions are, however, frequently non-veridical and fail to adequately capture hallucinations that are ‘indistinguishable from reality’ (high veridicality) or that ‘arise spontaneously’ (high spontaneity). To address this limitation, a framework has been proposed that couples DCNNs with deep generator networks (DGNs) and operationally defines hallucinations along three dimensions (veridicality, spontaneity, and complexity) in order to reproduce the qualitative differences among hallucinations of different aetiologies (Suzuki et al., 2024). Specifically, this work employs the DGN-AM architecture proposed by Nguyen et al. (2016). In this model, when generating an input that maximises the activation of a target layer in Classifier C (DCNN), a deep generative network G based on Dosovitskiy and Brox (2016) is coupled, and the statistical constraints on natural images (natural image prior) maintained by G secure veridicality. The GAN framework is used for training G, involving three components: Classifier C, Generator G, and Discriminator D (Figure 2A). This tripartite structure later serves as the departure point for the C × G × D framework of this paper, but in this section the focus is first on how the operations of C and G determine the qualitative differences among hallucinations.

The crux of this constructive logic is that ‘what kind of hallucination arises’ is described not simply as ‘noise has increased’ but in terms of the strength of the constraint (prior) and the dynamics of iterative optimisation. For instance, the hallucinations observed in neurodegenerative conditions (PD/DLB, etc.) have been understood against a backdrop of increased uncertainty in external input (diminished sensory precision), leading to heightened reliance on the internal model. Within predictive processing, this is discussed as ‘excessive reliance on priors’ (Teufel et al., 2015; Powers et al., 2017). In the model of Suzuki et al., precisely this condition is implemented as iterative reconstruction under strong constraints, yielding outputs with high veridicality (Suzuki et al., 2024). By contrast, psychedelic-type hallucinations correspond to conditions in which, as the REBUS (Relaxed Beliefs Under Psychedelics) model predicts, the precision weighting of higher-order priors is relaxed (Carhart-Harris and Friston, 2019); with the constraint (natural image prior) relatively loosened, abstract features of the classifier are excessively amplified and generated as complex, non-veridical patterns (Suzuki et al., 2024). Here, Huxley’s intuition of the ‘reducing valve’ can be computationally recast as the exposure of the classifier’s internal features accompanying constraint relaxation (systematised in Section 4).

Concretely, systematic manipulation of the presence or absence of the generator (DGN-AM vs. DCNN-AM), the target layer of the DCNN (low-level convolutional layers vs. high-level fully-connected layers), and the number of iterations confirmed a high degree of similarity with subjective ratings by patients in each aetiological group (Suzuki et al., 2024). This result demonstrates that the methodological cycle of computational phenomenology, in which first-person phenomenological data directly constrain model parameters and the model returns qualitatively valid output, can function empirically. Building on this constructive success, the next section integrates the operational variables identified (target layer, generative constraint, iterative process) into a unified formulation of the interplay among Classifier C, Generator G, and Discriminator D.

## 4 Objective functions as phenomenological descriptors

Section 3 showed that the features exposed by Classifier C and the constraint of Generator G’s natural image prior determine the qualitative differences among hallucinations (veridicality and complexity). The DGN-AM architecture surveyed in section 3.2 also includes GAN Discriminator D, but the significance of D for the phenomenology of altered states of consciousness has not yet been discussed. This section introduces this third component D alongside C and G, and examines in an integrated manner how objective functions (what is being optimised) and architectures (through what kind of interaction updates proceed) within the C × G × D framework jointly produce phenomenological differences. Section 4.1 below defines the framework and its epistemological premises; section 4.2–section 4.4 analyse each component; and section 4.5 situates the framework within existing neural computation theories.

### 4.1 The C × G × D framework

**Figure 3:**
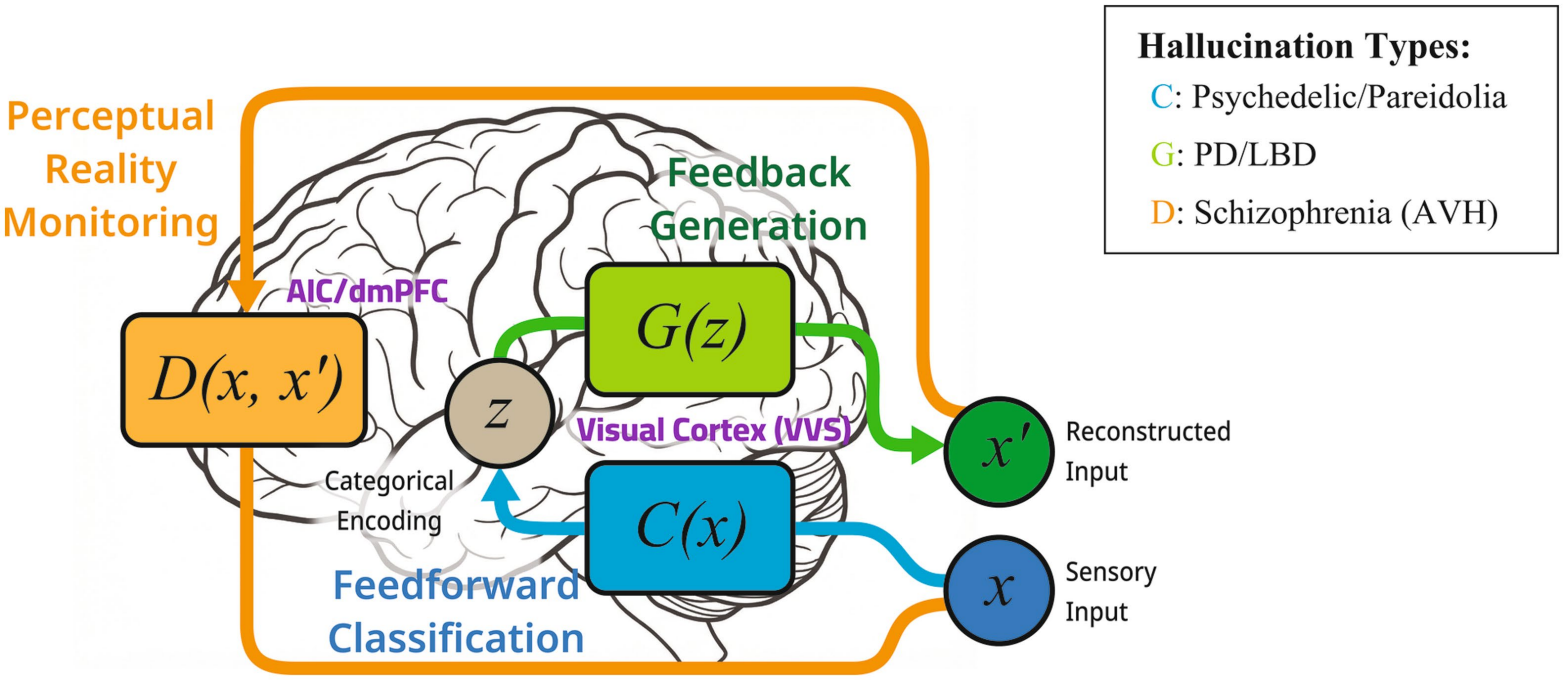
The C × G × D framework for altered states of consciousness. C (Classifier) performs feedforward classification in the ventral visual stream (VVS), encoding categorical information in higher-order areas (e.g., inferotemporal cortex, hippocampus). G (Generator) performs feedback generation through the same visual system, reconstructing visual input from these higher-level representations. D (Discriminator) performs perceptual reality monitoring in a frontal network including the anterior insula and dorsomedial prefrontal cortex (dmPFC) (Dijkstra et al., 2025). This architecture differs from Gershman (2019) ‘Generative Adversarial Brain’ framework in two respects: First, Gershman conceptualises both feedforward and feedback pathways as components of a single generator, whereas the present framework assigns the feedforward pathway an explicit classifier role (C), reflecting the distinct computational function of categorical recognition in the ventral stream, with only the feedback pathway serving as a generator (G); second, Gershman maps the discriminator onto the median anterior prefrontal cortex (aPFC), consistent with classical reality monitoring (Simons et al., 2017), whereas the present framework aligns D with the anterior insula–dmPFC network identified by Dijkstra et al. (2025) as implementing perceptual (rather than metacognitive) reality monitoring. The framework predicts three hallucination mechanisms: C-type (psychedelic hallucinations, arising from recession of G’s natural image prior and consequent exposure of C’s effective causes), G-type (PD/DLB visual hallucinations, arising from wrong-category generation with preserved veridicality), and D-type (schizophrenia/AVH, arising from source-monitoring failure where internally generated representations are misattributed as external).

With this overview in place, the computational roles of the three components are defined here explicitly (Figure 3). Classifier C is a trained deep convolutional neural network that hierarchically processes input images and extracts features effective for classification decisions (effective causes). The features that C exposes do not necessarily coincide with the visual features experienced in everyday perception (the structures and textures of natural environments), and this very ‘discrepancy’ carries phenomenologically significant information (details in section 4.2). Generator G is a deep generator network or GAN generator that, through a mapping from latent space (a lower-dimensional space of learned internal variables) to image space (generative prior), produces images that implicitly retain the statistical structure of natural images (natural image prior). The strength of the constraint imposed by G directly governs the veridicality of the generated output and corresponds to the presence or absence of a ‘taken-for-real’ quality in clinical hallucinations (details in section 4.3). Discriminator D is the evaluation module in a GAN that judges whether a generated output is ‘real’ or ‘fake’ and is positioned here as the computational correlate of Perceptual Reality Monitoring (Lau, 2019, 2022) in the brain. In this paper, the process that D subserves is primarily the judgement of source origin (external input vs. internal generation) (see section 4.4). D’s output threshold determines whether an internally generated representation is ‘adopted as real,’ corresponding to the alterations in sense of reality and confidence observed in hallucinations.

This tripartite C × G × D structure directly draws on the training architecture of Dosovitskiy and Brox (2016) introduced in section 3.2 (the co-training of Classifier C, Generator G, and Discriminator D) as a reinterpretation of computational mechanisms in the brain. Gershman (2019) ‘Generative Adversarial Brain’ addresses only the adversarial interaction between Generator G and Discriminator D, treating the feedforward and feedback pathways as components of a single generator; this paper differs in assigning Classifier C an independent computational role in the feedforward pathway (Figure 3). This separation yields differential predictions that neither Gershman’s two-component model nor the Strong Priors account (Corlett et al., 2019) can readily generate. In Gershman’s unified-generator framework, the feedforward pathway has no independent parameter, and the layer-specific geometric structure of psychedelic hallucinations remains unaccounted for. Similarly, the Strong Priors hypothesis attributes hallucinations to excessively precise priors within a single generative model but does not predict how targeting different hierarchical levels of the classifier produces qualitatively distinct visual patterns (geometric vs. object-like). The C × G × D framework makes this layer-dependent phenomenological variation an explicit, manipulable independent variable, and variations in C × G × D parameters generate different phenomenological profiles (see section 5.1).

This mapping should be understood not as a naive identification of the form ‘GAN = brain’ but as a division of labour among computational roles (generation, constraint, and endorsement). By rearranging sensory cortex, prefrontal cortex, and global networks according to the computational function each performs (generation, constraint, and endorsement), the framework renders the differences among altered states comparable not as ‘where something broke’ (lesion site) but as ‘how something went awry’ (failure mode: excess or deficit).

The correspondence between DCNNs/GANs and brain mechanisms proposed in this paper can be read at three distinct levels. (a) Structural analogy: a descriptive-level correspondence in which the computational division of labour between feedforward classification and the generative–discriminative interaction parallels the functional division between sensory cortex and prefrontal cortex. (b) Useful metaphor: a rhetorical device that renders complex neural processes comprehensible, as in ‘reducing valve’ or ‘forger and connoisseur.’ (c) Testable hypothesis: a falsifiable empirical claim to the effect that manipulation of each variable in the C × G × D framework yields quantitative predictions for specific psychophysical measures (reality-judgement threshold, confidence gradient, source-misattribution rate). The discussion in this paper intentionally moves between these three levels. The presentation of the theoretical framework (Sections 2–4) proceeds primarily at levels (a) and (b), while the experimental proposals (Section 5) stand explicitly at level (c). The reader is invited to distinguish the level at which each claim holds. Of particular importance is the fact that even if structural analogy (a) fails to hold, the experimental predictions (c) are independently testable; that is, even if the C × G × D framework is not a faithful image of the brain, if its operational variables systematically account for variance in behavioural measures, the framework is justified as a useful computational tool.

### 4.2 DCNN visualisation and effective causes

The core insight from DCNN visualisation research is that a network does not maintain a faithful internal copy of the external world; rather, it projects the input onto features that are ‘effective’ for the task (classification). As introduced in section 3.1, AM synthesises the input that maximises a target unit’s activation (Erhan et al., 2009), and subsequent refinements, including gradient-based saliency maps (Simonyan et al., 2014), natural image regularisation (Mahendran and Vedaldi, 2015), and Feature Visualisation (Olah et al., 2017), have systematised this approach.

In this paper, the features necessary and sufficient for classification, as exposed by these visualisation and inversion methods, are termed effective causes, a concept that can bridge to phenomenology. The notion of cause here carries no metaphysical commitment; it has the operational sense of an input feature that contributes to performing the classification task (cf. Olah et al., 2017). The significance of this concept is thrown into sharper relief by adversarial-examples research. DCNNs actively exploit not only features that humans regard as semantically important but also ‘non-robust features’ that are invisible to humans yet effective for classification, so that minuscule perturbations can drastically alter a DCNN’s classification result (Szegedy et al., 2013; Ilyas et al., 2019). This means that the ‘effective causes’ of a DCNN can in principle diverge from human perceptual categories, and when they are projected into input space through visualisation, patterns that are alien and ‘hallucinatory’ for human observers emerge. That DeepDream-type optimisation produces patterns resembling psychedelic phenomenology is precisely because these effective causes, normally suppressed in the human visual system, are projected into input space and foregrounded as appearances (Suzuki et al., 2017; Suzuki et al., 2024).

This framework of ‘excessive exposure of effective causes’ connects directly to a classical metaphor in consciousness research: the ‘reducing valve’ (Huxley, 1954). Swanson (2018) comprehensive review situates this concept within the lineage of ‘filtration theory’ tracing back to James, Myers, and Bergson, and identifies the central ambiguity: whether the ‘other side’ of the valve affords access to a broader ontological reality or to latent aspects of the mind.

Huxley (1954) original argument merits fuller exposition. Writing from his mescaline experience, Huxley proposed that the brain functions as a ‘reducing valve’ filtering ‘Mind at Large,’ admitting only the narrow trickle of information useful for biological survival. This is an ontological claim, presupposing a wider reality that exists independently of the brain’s filtering. The valve is a biological necessity: an organism perceiving the full range of possible experience would be overwhelmed and unable to act adaptively. The present paper also endorses the disinhibition account in which psychedelics ‘release’ the reducing valve, but differs regarding the nature of what is suppressed. In the C × G × D framework, what is revealed when the valve opens is not ‘Mind at Large’ in the ontological sense but the effective causes learnt by Classifier C, computational features that are normally suppressed behind G’s constraint during ordinary perception. On this account, what C has learnt through its classification objective is precisely the information useful for biological survival that Huxley’s valve was postulated to select. The idea that psychedelics reveal raw, unfiltered sensory input is intuitively appealing, yet the phenomenology of visual alteration argues against it. Psychedelic visual changes are neither amorphous nor random but exhibit specific, recurring patterns, including the amplification of meaningfulness and the foregrounding of lower-level visual features. This structured character is more consistent with the exposure of a classifier’s hierarchically organised effective causes than with unfiltered access to sensory data. The abstract principle that Swanson identifies as running through successive psychedelic theories, a disruption of a universal brain process that normally constrains perception, emotion, cognition, and self-sense, connects directly with this computational reading. The C × G × D framework thus provides a computational account of what changes in the perceptual process without requiring Huxley’s metaphysical assumption; whether a ‘wider ontological truth’ exists beyond the effective causes of the classifier is a question the framework leaves open.

This reformulation is given concrete form through its junction with the REBUS model and the Entropic Brain hypothesis. The REBUS model holds that under psychedelics the precision weighting of higher-order priors is relaxed, thereby ‘liberating’ signals and exploration from lower levels (Carhart-Harris and Friston, 2019). The Entropic Brain hypothesis posits that increased brain-activity entropy corresponds to greater diversity and richness in subjective experience, characterising the ordinary waking state as ‘secondary consciousness’ (a state in which the default-mode network (DMN) stably maintains the self-model and entropy is suppressed) and the psychedelic state as ‘primary consciousness,’ a state in which entropy rises (Carhart-Harris et al., 2014). The DCNN visualisation framework makes the computational substance of this ‘liberation’ explicit. The relaxation of higher-order priors that REBUS describes corresponds, in the terms of this paper, to the release of the selective constraint that Classifier C imposes on input, that is, the opening of the ‘reducing valve.’ Normally, C’s effective causes are hidden behind classification decisions; when the precision weighting of priors decreases, these effective causes are excessively projected into input space, foregrounded as geometric patterns and excessive pattern recognition characteristic of the psychedelic state. The finding that decreased alpha-band power in the posterior cingulate cortex (PCC) correlates with ego-dissolution experience (Carhart-Harris et al., 2014; cf. Muthukumaraswamy et al., 2013; Lebedev et al., 2015) suggests that this ‘release of the reducing valve’ is measurable as a modulation of specific neural oscillatory dynamics.

Connecting these findings with DCNN visualisation, what is foregrounded in the psychedelic state is not ‘the external world itself’ but rather a condition in which the effective causes by which the classifier carves up the world are ‘drawn back’ into input space without suppression. In other words, the ‘other side’ of the reducing valve is modelled not as an augmentation of objective reality but as the excessive exposure of features effective for classification. This excessive exposure is closely related to the enhancement of meaningfulness (feeling of meaningfulness; Preller et al., 2017) and aberrant salience (Kapur, 2003; Wiessner et al., 2023) widely reported in psychedelic experience. Within the C × G × D framework, the excessive exposure of C’s effective causes is positioned as the computational basis for conferring aberrant salience upon neutral inputs, and the patterns generated by DeepDream function as an externalisation of this process (Keshavan and Sudarshan, 2017).

### 4.3 Generative models and the natural image prior

As discussed in section 3.2, when projecting C’s effective causes into input space, the presence or absence of Generator G decisively determines phenomenological quality. The strength with which G’s natural image prior constrains C’s effective causes governs the veridicality of the generated output: under strong G-constraint, veridical hallucinations (the neurodegenerative type) come to the fore; under weak G-constraint, non-veridical patterns (the psychedelic type) are foregrounded (Suzuki et al., 2024). This section examines the theoretical implications of this asymmetry in greater depth.

To elaborate on this contrast, at the level of structural analogy (level (a) in section 4.1): the veridical images generated by G can be regarded as a computational state that is structurally parallel to what we see in ‘normal’ perceptual experience; that is, normal perception can be described as a state in which G stably generates an internal representation consistent with sensory input and effective causes thereby recede into the background (this parallel is, however, restricted to the single dimension of veridicality constraint; a full identification of the two is not claimed). Under strong G-constraint, perception becomes ‘transparent’—the representational medium itself is not experienced as such (Metzinger, 2003)—and perceptual presence (Seth, 2014), the quality of experiencing perceptual content as ‘really existing here and now as part of the world’, is secured. When G’s constraint recedes, transparency and the sense of presence both waver, and effective causes that are normally hidden in the background are foregrounded. Understanding psychedelic perception in this manner is one of the central claims of this paper. Computational evidence for this view is provided by Reichert et al. (2013), who used a Deep Boltzmann Machine to model Charles Bonnet syndrome and showed that when bottom-up constraint is lost due to sensory input deprivation, the learned generative model spontaneously produces veridical hallucination-like patterns through homeostatic compensation. This is an instance in which G’s generative prior comes to the fore when external input to C is lost, and is understandable as a computational demonstration of hallucination under a G-strong condition (the neurodegenerative type).

As discussed in section 3.2, in the Dosovitskiy and Brox (2016) model, G is trained to reconstruct images using the internal representations of C as input, and G is paired with and constrained by C. Within the GAN framework, G and D are co-trained through adversarial learning. This means that two computational functions (generation and reality monitoring) are simultaneously implemented in an interdependent manner (Gershman, 2019), offering an important suggestion for thinking about the relationship between perceptual generation and reality monitoring in the brain. The two may be understood not as independent modules but as functions differentiated from a common learning and developmental process.

In sum, the asymmetry between C and G can be stated as follows. Under sufficiently unregularised optimisation, C’s effective causes take on a psychedelic quality, much as a human visual system under the influence of a hallucinogen foregrounds the ‘raw material’ of visual processing. G superimposes natural image statistics on these effective causes, conferring veridicality and realising the ‘transparency’ of normal perception. This asymmetry furnishes an architecture-level ground for the correspondence, within the C × G × D framework, that ‘psychedelic perception = the exposure of C’s effective causes through the recession of G’s constraint.’

### 4.4 Discriminator and perceptual reality monitoring

The preceding sections discussed the mechanism by which Classifier C exposes effective causes (section 4.2) and the mechanism by which Generator G controls veridicality through the constraint of a natural image prior (section 4.3). The remaining question concerns the computational process by which a generated representation is adopted as real, or fails to be so adopted. On this question, the architecture of GANs provides a suggestive structure. In a GAN, Generator G is trained as a ‘forger’ that produces samples imitating the distribution of training data, while Discriminator D serves as a ‘connoisseur’ that judges whether a sample is genuine (derived from the training data) or counterfeit (produced by G) (Goodfellow et al., 2014). When adversarial training converges, G’s products become indistinguishable from the genuine article and D reaches the limits of its discriminative capacity. Translating this ‘forger and connoisseur’ dynamic into neural computation, Discriminator D can be interpreted as a computational implementation of reality monitoring that judges ‘whether this representation originates from external input or from internal generation.’ It is the threshold control of this ‘reality adoption’ that constitutes the role of the third component, Discriminator D.

‘Reality monitoring’ encompasses multiple levels. Classical Reality Monitoring (Johnson and Raye, 1981) refers to metacognitive judgements concerning whether a given memory derives from actual experience or from imagination, that is, retrospective source attribution. As the neural substrate of this level, the anterior prefrontal cortex (aPFC) has been identified as the core region (Simons et al., 2017), considered to support primarily metacognitive processes involved in memory source judgements. By contrast, what Lau (2022) terms Perceptual Reality Monitoring (PRM) is the process by which an ongoing percept is evaluated in real time as originating from external input or from internal generation, an operation at a different computational level from classical reality monitoring. The Discriminator D in this paper operates at this PRM level.

A review has demonstrated that no single factor (signal strength, precision, contextual expectation) can reliably distinguish the external from the internal origin of a representation (Dijkstra et al., 2022). Concrete neural substrates for this PRM have been provided by Dijkstra et al. (2025), who showed that the fusiform gyrus encodes sensory signal strength as a continuous ‘reality signal,’ that the anterior insula cortex serves as the core node that converts this signal into a reality judgement, and that a frontal network including the dorsomedial prefrontal cortex (dmPFC) transforms this continuous signal into a binary categorical decision. This anterior insula–dmPFC network constitutes the most appropriate candidate neural substrate for the PRM that D implements in this paper. These findings imply the theoretical necessity of a dedicated monitoring mechanism that goes beyond individual sensory properties. Gershman (2019) ‘Generative Adversarial Brain’ provides a computational framework that addresses precisely this PRM-level requirement. In this model, the discriminator’s endorsement of perceptual content as ‘real’ constitutes the condition for subjective experience, and certain types of hallucination are formalised as the event in which the discriminator erroneously endorses an internally generated product as ‘reality.’ Gershman’s discriminator performs not retrospective source attribution of memories but real-time authenticity judgement during ongoing perception, which directly corresponds to Lau’s PRM.

The C × G × D framework draws a key distinction regarding the computational mechanisms of hallucination in schizophrenia. Whereas psychedelic-type hallucinations are described as the retreat of G’s constraint and the exposure of C’s effective causes (section 4.2–section 4.3), hallucinations in schizophrenia are better understood as arising from an abnormality of Discriminator D, that is, of reality monitoring. The crux of the problem lies not in the generation of perceptual content (G) but in the dysfunction of the judgement process (D) that erroneously endorses internally generated representations as ‘real.’ Hallucination in schizophrenia is associated with dysfunction of frontal monitoring systems, including hypoactivation in medial prefrontal cortex (Garrison et al., 2017). This framework is also applicable to auditory verbal hallucinations (AVH). According to Frith (1992) efference copy model, AVH arise from impaired corollary discharge accompanying the generation of inner speech, such that self-generated verbal signals are misattributed to an external source. Within the C × G × D framework, this is formalised as a failure of D’s source monitoring with respect to internally generated representations produced by G, specifically a lowering of the threshold at which internal products are erroneously endorsed as ‘external input’ (Blakemore et al., 2002; Garrison et al., 2017).

The computational mechanisms underlying hallucinations in schizophrenia remain debated. In C × G × D terms, the primary locus of dysfunction is D: a lowered threshold at which internally generated representations are erroneously endorsed as real. The role of generative priors (G) in schizophrenia remains a matter of active debate in Bayesian modelling. The strong priors account holds that abnormally precise prior beliefs override sensory evidence (Corlett et al., 2019; Powers et al., 2017); the weak priors account proposes that imprecise priors fail to constrain perception (Fletcher and Frith, 2009); and the circular inference framework models hallucinations as arising from reverberation between excitatory loops, rather than from aberrant prior strength per se (Jardri and Denève, 2013; Jardri et al., 2017). The C × G × D framework adopts the position that D-dysfunction is the defining mechanism of this hallucination type, while acknowledging that G may be concurrently modulated. Table 1 accordingly marks G as ‘variable (debated)’ for the D-type configuration, as the current evidence does not warrant a single commitment.

**Table 1:** Summary of C × G × D parameter configurations for different perceptual states and hallucination types.

Configuration	C (Effective causes)	G (Generative prior)	D (Reality threshold)	Phenomenological output
C-type (Psychedelic)	High exposure	Weak	Normal–low	Geometric patterns, aberrant salience
G-type (Neurodegenerative)	Normal	Strong (wrong category)	Normal	Veridical formed hallucinations
D-type (Schizophrenia/AVH)	Normal	Variable (debated)	Dysfunctionally low	Source misattribution
Normal perception	Suppressed	Strong (correctly constrained)	Calibrated	Transparent, veridical experience

What computational process D ultimately implements requires further specification. In Seth (2014) framework, the ‘reality-likeness’ of perception is differentiated into multiple dimensions. Perceptual Reality refers to the existence of perceptual phenomenology itself, the property that distinguishes genuine percepts from mere thoughts or imagery, and it is precisely this level that Perceptual Reality Monitoring evaluates: the judgement of whether a representation originates from external input or from internal generation (Lau, 2022). By contrast, perceptual presence, the phenomenon that Seth (2014) identifies with Subjective Veridicality, is the phenomenological quality of experiencing perceptual content as part of the real world, and depends on counterfactually rich generative models grounded in sensorimotor contingencies and affordances (Seth, 2014; Suzuki et al., 2023). This paper adopts PRM as the primary computational process that D subserves, which operates at Seth’s level of Perceptual Reality, a process at a different level from perceptual presence (= Subjective Veridicality). Because perceptual presence possesses a multisensory foundation (Suzuki et al., 2023), it is positioned here as a secondary measure within the present paper’s vision-centred experimental proposals. As Dijkstra et al. (2022) note, the experience of ‘feeling real’ and the belief that ‘this is real’ can dissociate, and PRM operates at a level further distinguished from both of these. Although D in this paper primarily operates at the level of PRM-type source monitoring, its relationship to perceptual presence remains an open empirical question, and the experimental design proposed in section 5.1 is also intended to function as a dual test that can discriminate between these two levels.

The threshold mechanism of D bears a structural analogy to multisensory causal inference (Körding et al., 2007; Shams and Beierholm, 2010), in which the brain infers whether sensory signals share a common cause, integrating them if the posterior probability exceeds a threshold. D’s reality-monitoring threshold operates at a comparable decision boundary. However, while causal inference concerns common versus independent causes of sensory signals, D concerns the internal versus external origin of a representation, aligning it more closely with the comparator model of sense of agency (Frith et al., 2000; Blakemore et al., 2002). The causal inference analogy nonetheless remains relevant for bodily self-consciousness: in the rubber hand illusion (Botvinick and Cohen, 1998), body ownership arises from inferred common causation of coincident visual and tactile signals, interpretable as D operating over multisensory representations. This bridges to the ego-dissolution discussion in section 5.2, where lowering D’s threshold in the multisensory domain would correspond to a dissolution of the self–other boundary.

### 4.5 Connections with existing neural computation theories

Each component of the C × G × D framework possesses connection points with existing theories of neural computation. AM of Classifier C is an operation that holds all network weights fixed and updates only the input by gradient ascent to maximise activation at a target layer; by releasing the constraint of sensory input and driving generation solely by the internal model, it functionally illustrates the principle of predictive processing (Suzuki et al., 2017). This also serves as a computational illustration of the Strong Priors hypothesis of Corlett et al. (2019), which holds that abnormally strong prior beliefs drive hallucinations. Regarding the backpropagation (the standard algorithm that adjusts network weights by propagating error gradients backward through layers) on which AM relies, recent research has shown that it can be approximated using only local Hebbian rules (Whittington and Bogacz, 2017; Millidge et al., 2022), increasingly securing the biological plausibility of this operation.

The foregoing concerned C’s standalone implementability; turning now to the coupling of C and G, the relationship is intimately related to the distinction between ‘fast’ and ‘slow’ processing in vision. The visual system extracts a global summary (gist) of the scene through an initial feedforward sweep within the first 100–150 milliseconds (Thorpe et al., 1996; Oliva and Torralba, 2006), and subsequently achieves detailed object recognition and figure-ground segregation through recurrent processing (Lamme and Roelfsema, 2000). Bar (2003) proposed a mechanism whereby low spatial frequency information is rapidly projected to the orbitofrontal cortex, serving as a top-down predictive signal that facilitates detailed recognition. The Hybrid Predictive Coding model of Tschantz et al. (2023) computationally formalises this two-stage processing, unifying initial feedforward processing as amortised inference and subsequent recurrent refinement as iterative inference under variational free energy. In the C × G × D framework, C’s rapid feedforward classification corresponds to amortised inference and G’s iterative generative process corresponds to iterative inference; this paper predicts that the transition from ‘fast recognition’ to ‘slow generation’ governs the temporal dynamics of hallucination generation.

Regarding the adversarial interaction between G and D, that is, the coupling of generation and discrimination, GAN-type inference extensions provide important precedents. Bidirectional GAN architectures (Dumoulin et al., 2017; Donahue et al., 2017) have shown that Discriminator D functions not only as a forgery detector but also as a feedback signal that improves inferential quality; more recently, the Generative Adversarial Inference framework of Jeon et al. (2025) incorporates uncertainty handling and derives quantitative predictions of attraction and repulsion biases in perception, indicating that GANs are not merely a metaphor but a framework capable of yielding predictions for concrete perceptual phenomena. It should be noted that implementation in the brain need not be restricted to GAN-style adversarial learning; alternative implementations that operate entirely through local learning rules without recourse to backpropagation, such as the Forward-Forward algorithm (Hinton, 2022), are also possible in principle.

Finally, it is worth making explicit how the C × G × D framework, whose general positioning relative to FEP-centred CP was outlined in section 2.3, relates to specific programmes. The framework realises Ramstead et al. (2022) ‘generative passages’ while shifting the locus of phenomenological difference from ‘what the model infers’ to ‘how the model is built.’ It complements da Costa et al. (2024) information-geometric quantification of phenomenological distance by identifying the structural axes generating those distances, and extends Sandved-Smith et al. (2025) domain-specific application to aetiologically distinct hallucinations via an independently manipulable DCNN/GAN architecture. Beckmann et al. (2023) argued for treating deep learning as an autonomous source of phenomenological inquiry, bracketing neurophysiology; the present framework can be viewed as a worked application of their programme to the phenomenological differentiation of visual hallucinations.

### 4.6 Summary

From the foregoing analysis, the C × G × D framework predicts three hallucination mechanisms (Figure 3). C-type hallucinations (psychedelic) are described as the recession of G’s natural image prior and the consequent excessive foregrounding of C’s effective causes (section 4.2–section 4.3). G-type hallucinations (neurodegenerative, such as PD/DLB visual hallucinations) correspond to the generation of veridical images of the wrong category while G’s prior is preserved, explaining the high-veridicality hallucinations observed in Parkinson’s disease and dementia with Lewy bodies. This prediction is already partially supported by the fact that images generated via DGN-AM showed high similarity to actual patients’ subjective ratings (Suzuki et al., 2024). D-type hallucinations (schizophrenia/AVH) are described as dysfunction of D’s reality monitoring, in which internally generated representations are misattributed as external (Frith, 1992; Garrison et al., 2017), a process structurally parallel to a lowering of the GAN discriminator’s threshold. This prediction is testable as reduced performance on source-monitoring tasks in schizophrenia patients and is incorporated into the psychophysical experiment in section 5.1 as individual differences in D-threshold. These three types are not mutually exclusive; in clinical presentation they may co-occur as combined modulations, but the separation of each axis as an independently operable variable constitutes the methodological advantage of the C × G × D framework. Table 1 summarises these configurations.

Section 5 translates this three-axis map into experimental manipulations.

## 5 Discussion

This section translates the C × G × D framework into experimental predictions (section 5.1), extends the reducing-valve reformulation to ego dissolution (section 5.2), and addresses limitations (section 5.3).

The central claim of this paper is methodological. Qualitative differences among altered states of consciousness should not be redescribed as differences in ‘representational content.’ Rather, they emerge from variations in the objective functions, constraints, and gains of a framework comprising generation, discrimination, and classification. This redescription provides a scaffold for deriving testable predictions.

### 5.1 Phenomenology as stimulus generation

**Figure 4:**
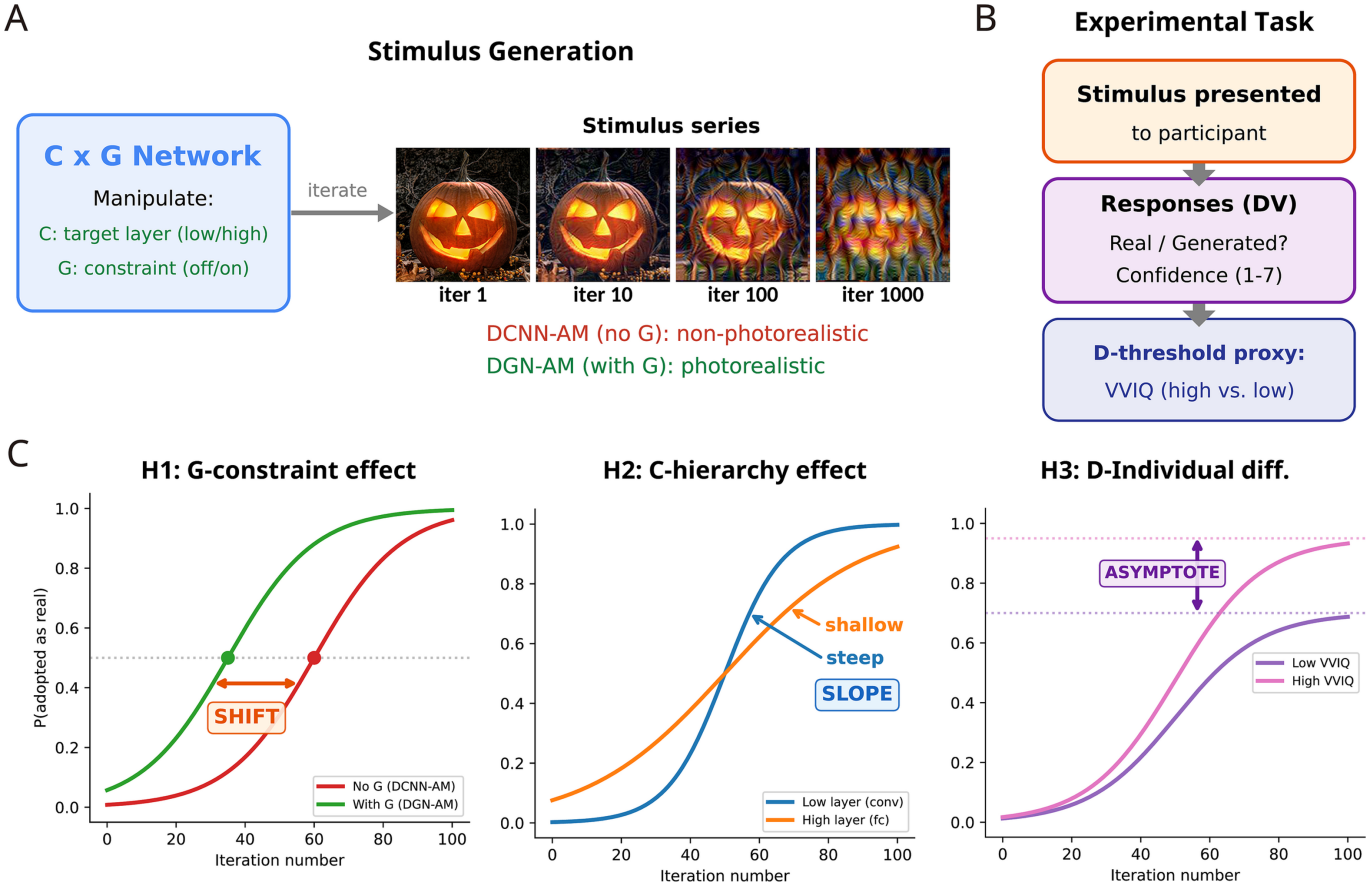
Proposed experimental pipeline. **(A)** Stimulus generation via the C × G network: independent manipulation of C’s target layer (low vs. high), G’s constraint (absent [DCNN-AM] vs. present [DGN-AM]), and iteration number. The example stimulus series shows DCNN-AM (no G) at a lower convolutional layer. **(B)** The experimental task: stimuli are presented to participants, who provide reality judgements (real/generated) and confidence ratings. D’s threshold is not implemented in the network but operationalized through task manipulations and individual differences in vividness of visual imagery (VVIQ). **(C)** Predicted psychometric curves for the three hypotheses: H1 (G-constraint effect, leftward shift with G present), H2 (C-hierarchy effect, shallower slope at higher target layers), and H3 (D-individual differences, higher asymptote for high-VVIQ participants).

First-person reports have conventionally been treated as ‘dependent variables with large measurement error.’ With the introduction of generative models, however, first-person reports are instead transformed into constraints for identifying the stimulus space. The role of the network models in the experimental design proposed below must be clarified: they serve as stimulus generation devices, not as models of the participant’s decision-making process. C and G are used to systematically generate visual stimuli by manipulating computational parameters (target layer, generator constraint, iteration number), while participants’ behavioural responses (reality judgement, confidence, source attribution) provide the dependent variables. D’s threshold is not directly implemented in the network at test time but is operationalised through task manipulations and individual differences (see below; Figure 4). As established in section 4.2, AM exposes the classifier’s effective causes rather than a faithful image of the external world, and representational inversion quantifies what information a representation retains (Dosovitskiy and Brox, 2016). Integrating these two methods, phenomenological data can be embedded not as ‘impressions described in language’ but as the optimisation objective for a stimulus-generation system that includes Generator (G), Discriminator (D), and Classifier (C). In other words, the task of neurophenomenology is reframed: from ‘correlating reports with brain activity’ to ‘identifying the computational conditions under which stimuli matching reports are generated.’

This strategy of ‘incorporating the first person as a model constraint’ is being systematised in recent work. The ‘triple braid’ methodology reviewed in section 2.1 (Sandved-Smith et al., 2025), along with Predictive-Coding-Inspired Variational recurrent neural network models that formalise transitions between meta-attentional states as precision-weighting modulation (Idei et al., 2025), demonstrate that computational phenomenology is a general research programme not limited to any specific model. The iterative-optimisation-based psychophysical experiment proposed here is situated within this established methodological lineage.

The minimal experimental design can be specified as follows. Three independent variables are manipulated, one along each axis of the C × G × D framework. On the C-axis, the target layer of the DCNN (GoogLeNet Inception v1, the architecture employed in the original DeepDream and Hallucination Machine studies) is varied between low-level convolutional layers and high-level fully-connected layers, alongside the intensity of Activation Maximisation iterations. On the G-axis, the presence and constraint strength of the generator is varied (DCNN-AM [no G] vs. DGN-AM [with G]). On the D-axis, task manipulations (confidence instructions, time pressure) and individual differences (Vividness of Visual Imagery Questionnaire (VVIQ) scores) serve as proxies for discriminator-threshold variation. The dependent variables are: real/generated binary judgement, confidence (continuous scale), and source attribution (external/internal). Because VVIQ is a metacognitive self-report measure that may covary with response bias and absorption tendency, the experimental protocol should additionally include signal-detection-theory-based indices (criterion c and d’) to isolate D-threshold variation from general response tendencies.

The function to be estimated is the psychophysical curve of ‘probability of adopting as real’ as a function of iteration number, and the key question is how the three variables (C layer, G presence, D-threshold) modulate the shift, slope, and asymptote of this curve.

In addition to this methodological grounding, the iterative-optimisation-based psychophysical experiment proposed here connects with existing experimental paradigms in visual science. For instance, experiments that measure the threshold for ‘what is seen’ using images from which noise has been progressively removed or morphing images are, in essence, operationally defining ‘the threshold of the human internal discriminator.’ The C × G × D framework generates testable predictions through the systematic manipulation of this discriminator threshold. Concretely, by presenting participants with stimulus series in which the number of Activation Maximisation iterations is systematically varied (two conditions: DeepDream-type and DGN-AM-type) and measuring reality judgement (real/generated), confidence, and source attribution, the ‘sensitivity curve’ of the discriminator can be estimated at the behavioural level.

The source-mixing model (Dijkstra and Fleming, 2023) indicates that signals from perception and mental imagery are mixed at the level of subjective intensity, and that the integrated signal is experienced as real when it exceeds a ‘reality threshold.’ From the C × G × D framework, three hypotheses are derived regarding the conditions under which this threshold is crossed. Hypothesis 1 (G-constraint effect): When Generator G is present (DGN-AM condition), the psychometric curve shifts leftward compared to the condition without G (DCNN-AM); that is, generated images are adopted as ‘real’ at fewer iterations. This follows directly from G’s natural image prior, which enhances veridicality and thereby makes it easier for the output to exceed D’s reality threshold. Hypothesis 2 (C-hierarchy effect): When higher-level fully-connected layers of the classifier are targeted, the slope of the psychometric curve becomes shallower compared to conditions targeting lower-level layers. The rationale is that as effective causes become more semantically complex, they are harder for D to distinguish from genuine perceptual input, reducing the discriminability of real/generated judgements. Hypothesis 3 (D-individual differences): The high-VVIQ group exhibits a higher asymptote on the psychometric curve than the low-VVIQ group. Vivid imagers, having a lower reality-adoption threshold for D, are more readily inclined to adopt internally generated products as ‘real.’ This prediction is consistent with the vulnerability of reality monitoring in vivid-imagery holders reported by Dijkstra and Fleming (2023).

If all three hypotheses are rejected, this would demonstrate that there is no systematic correspondence between the operational variables of the C × G × D framework and behavioural measures, constituting a falsification of the framework’s predictive utility. This experiment also functions as a dual test for the unresolved question raised in section 4.4: whether D primarily implements PRM-type source monitoring or also captures a broader mechanism related to perceptual presence. If Hypotheses 1–3 are confirmed while presence measures remain unaffected, D’s primary computational role would be identified as PRM-type source monitoring.

This experimental framework also connects directly to clinical application in the form of computational profiling. A patient explores generated images that approximate their own hallucinations using parameters such as G’s constraint (natural image prior), C’s target layer (degree of effective-cause exposure), and D’s threshold (stringency of reality adoption), feeding back a subjective match score; in this way, the symptom is externalised from ‘a verbal report’ to ‘a vector of operational variables’, functioning as a computational profile that precedes diagnostic categories.

### 5.2 Reinterpreting the reducing valve

Section 4 reformulated the ‘opening’ of the reducing valve as a composite of two independently manipulable variables: increased exposure of C’s effective causes and lowered D adoption threshold. This section extends that reformulation to the phenomenology of ego dissolution. According to the REBUS model, when relaxation of prior precision reaches the apex of the functional hierarchy—beliefs bearing on self-identity and ego—the result is experienced as ego dissolution (Carhart-Harris and Friston, 2019). The DMN–PCC correlations discussed in section 4.2 provide the neural locus for this process. Within the C × G × D framework, the experience of a ‘unified self’ in the ordinary waking state is structurally parallel to G’s veridical generation in vision: the diverse elements constituting the self (somatosensory self, interoceptive self, narrative self; Gallagher, 2000) are integrated into a ‘transparent whole’ by the higher-order generative process maintained by the DMN.

Drawing on the transparency/presence distinction established in section 4.3, the C × G × D framework makes two predictions about ego dissolution. G’s constraint retreat corresponds to a breakdown of transparency: the normally invisible constitutive processes of the self become foregrounded. Extending the reducing-valve reformulation of section 4.2 to the domain of selfhood, what is exposed here is not raw self-experience but the effective causes of a hierarchical self-model integrating multisensory signals into a unified sense of self. The holistic ego dissolves into its constituent components. D’sthreshold lowering corresponds to a modulation of perceptual presence: the sense that the self’s existence ‘feels real’ diminishes. This dual modulation permits an operational account of the qualitative difference between ‘incomplete ego dissolution’ and ‘complete ego dissolution,’ as predicted by REBUS (Carhart-Harris and Friston, 2019). In incomplete ego dissolution, G’s constraint partially retreats and constituent elements of the self are foregrounded, but D’s threshold has not lowered sufficiently; the discrepancy between the exposed constituents and the higher-order expectation of a unified self is experienced as anxiety. In complete ego dissolution, both G and D recede substantially: the constituent elements of the self are foregrounded and, simultaneously, the judgement of ‘whether this is self or not’ itself ceases.

The Ego-Dissolution Inventory (EDI; Nour et al., 2016) measures ego dissolution as a unidimensional construct, but its item content encompasses both the breakdown of self–other boundaries and the experience of cosmic unity. Moving from structural analogy to testable hypothesis (level (c) in section 4.1), the C × G × D framework predicts that these two item types track different axes: boundary-dissolution items should selectively correlate with G-constraint retreat (breakdown of the self’s transparency), while unity items should selectively correlate with D-threshold modulation (alteration in reality monitoring of the self–world boundary). Through the connection with the D-threshold psychophysical curve proposed in section 5.1, a quantitative prediction also becomes possible: individual differences on the EDI total score should correlate with the magnitude of D-threshold shift—the greater the ego-dissolution tendency, the lower D’s reality-adoption threshold, and the more readily internal products are adopted as ‘real.’

An epistemological limitation must be acknowledged. The C × G × D framework of this paper has focused on the level of Perceptual Reality Monitoring, which distinguishes perception from imagination. This focus is a choice made to secure operational clarity, but the C × G × D framework simultaneously admits of extension in two directions. First, given that the original Reality Monitoring (Johnson and Raye, 1981) addressed metacognitive source judgements, one can envisage a nested structure in which a perceptual-level D subserves PRM-type source monitoring while a higher-order, metacognitive-level D′ judges the origins of beliefs and memories. This higher-order D′ corresponds to the Doxastic Reality level discussed in section 5.3, where the failure of belief evaluation constitutes Factor 2 of Coltheart (2010) two-factor account of delusions. Second, extension to modalities beyond vision (interoception, proprioception) is also theoretically possible. These extensions are most acutely demanded by the phenomenology of ego dissolution. The experience of the collapse of the very distinction between subject and object, and the transformation of pre-reflective self-awareness (minimal self), as discussed by Zahavi (2005, 2018), cannot be captured at the single level of visual PRM. However, in a hierarchically extended framework, by combining the decomposition of self-subcomponents (G-constraint retreat) with multi-level modulation of reality attribution (threshold changes at both D and D′), the possibility arises that these phenomena too could be brought within the framework’s scope. This extension challenge, alongside the integration with enactive embodiment discussed in section 5.3, indicates the direction for future development of the C × G × D framework.

### 5.3 Limitations and next steps

As discussed in section 4.5, each component can be situated within existing theories of neural computation, but whether the C × G × D framework exists in the brain as ‘three modularly separated agents’ or as distinct computational phases within a single predictive-coding network remains an open question. In addition, implementing the experiments proposed in section 5.1 requires careful attention to risks associated with VR/immersive stimuli (exclusion criteria for psychiatric history, debriefing protocols, and pre-registration are essential).

The status of neuromodulators also warrants clarification. Although the C × G × D framework does not directly model neuropharmacology, it possesses connection points through which neuromodulatory effects can be translated into operational variables of the framework. For instance, serotonin 5-HT2A agonists (classical psychedelics) can, within this framework, be interpreted as exerting the following composite effects: (i) given the dense expression of 5-HT2A receptors on deep layer-V pyramidal cells (Celada et al., 2013), these agonists produce gain modulation at intermediate-to-higher cortical layers, corresponding to amplification of effective causes at specific layers of C (the classifier); (ii) 5-HT2A activation produces functional modulation of thalamo-cortical loops (Preller et al., 2019), which is translatable as modulation of the feedback coupling between G and D; (iii) 5-HT2A expression in the prefrontal cortex is interpretable as the neurochemical basis for fluctuations in D’s (discriminator = reality monitoring) threshold. Similarly, dopaminergic precision modulation can be situated in the context of D-threshold fluctuation, and the raising of D’s threshold by antipsychotics serves as a connection point to clinical pharmacology. These correspondences are presented here not as validated claims but as hypotheses generated by the C × G × D framework and awaiting validation through neuropharmacological experiment.

A clarification is also warranted regarding the relationship between the reducing-valve reformulation proposed in section 4.2 and existing computational accounts. The REBUS model (Carhart-Harris and Friston, 2019) describes the opening of the reducing valve as relaxed precision weighting on hierarchical priors, and the neural-field model of Bressloff et al. (2001) derives geometric form constants from V1 architecture without recourse to DCNN vocabulary. The present framework does not supplant these accounts but adds a complementary level of description: it specifies what is exposed when the valve opens (the effective causes of a classifier, whose visual structure depends on the targeted hierarchical level) and how the exposed content is evaluated (through D’s reality-monitoring threshold). This layer-dependent specification of hallucinatory content, that targeting different hierarchical levels of C produces qualitatively distinct patterns (geometric at lower layers, object-like at higher layers), constitutes a prediction that neither precision-weighting accounts nor neural-field models generate in their current form, and is directly testable in the experimental design of section 5.1.

The C × G × D framework addresses perceptual experience and does not extend straightforwardly to delusions. Delusions involve the formation and maintenance of beliefs that resist counterevidence, a process operating at a cognitive level beyond perceptual reality monitoring. Coltheart (2010) two-factor theory of delusions provides an instructive point of comparison. Factor 1, the anomalous experience, can be characterised within the C × G × D framework as arising from abnormalities in C (distorted effective causes yielding aberrant perceptual features) or G (a biased generative prior producing contextually inappropriate content). Factor 2, the failure to reject the resulting aberrant belief, is related to D but operates at a cognitive level higher than the Perceptual Reality Monitoring that D implements in this paper. Suzuki et al. (2023) distinguished Doxastic Reality, the belief-level endorsement that a state of affairs obtains in the world, from perceptual-level reality judgement; Factor 2 plausibly operates at this doxastic level, involving hypothesis evaluation and explanatory coherence that the current C × G × D architecture does not model. Delusions whose content is propositional rather than perceptual (grandeur, persecution, reference) would accordingly require an extension of D to this higher-order belief-evaluation process. This extension corresponds to the hierarchically nested D′ envisaged in section 5.2.

As for non-visual hallucinations, auditory verbal hallucinations have already been addressed within the framework as a failure of D’s source monitoring (section 4.4). The C × G × D architecture is in principle modality-general: classification, generation, and discrimination are computational roles not inherently restricted to vision. However, the current implementation relies on visual DCNNs and image-domain GANs, and extension to somatic, interoceptive, or proprioceptive modalities would require substituting these with modality-appropriate architectures, a direction that also bears on the extension to bodily self-consciousness and ego dissolution discussed in section 5.2. Systematic generalisation of the framework beyond the visual domain remains a task for future work.

More broadly, the title of this paper refers to ‘altered states of consciousness,’ and it is important to delineate the framework’s scope. A recent consensus taxonomy (Cardeña et al., 2025) identifies eight categories of altered states, of which the C × G × D framework directly addresses the imaginary/fantasy/visionary category (psychedelic, neurodegenerative, and schizophrenic hallucinations as C-type, G-type, and D-type configurations). It partially addresses altered identity and unity/mystical states through the ego-dissolution extension in section 5.2. The remaining five categories (proto and transitional, delirium, minimal to no awareness, experiential detachment, and enhanced physicality) fall outside the current scope, as they involve either global alterations in arousal that the framework does not model, or dimensions of embodiment and agency that would require integration with enactive approaches discussed below. The C × G × D framework is thus best understood not as a general theory of altered states but as a targeted tool for the computational phenomenology of perceptual altered states, with potential extensions to self-related states via the G–D modulation proposed in section 5.2.

The theoretical tension with enactivism noted in section 2 is a further limitation that this paper has not fully resolved. The bodily engagement, constitutive coupling with the environment, and sensorimotor contingencies emphasised by the enactive approach rest on a view of cognition fundamentally different from the ‘input → internal representation → output’ information-processing pipeline presupposed by this paper’s C × G × D framework (Thompson, 2007; Di Paolo et al., 2017). As Beckmann et al. (2023) proposed (section 2.2), reinterpreting network learning as the sedimentation and reactivation of habits opens a path towards incorporating enactive insights, but this integration lies beyond the scope of the present paper. This limitation also raises an epistemological question concerning the methodological character of the paper itself. The approach taken here employs the architecture and objective functions of computational models as ‘phenomenological descriptors,’ and unlike Varela’s neurophenomenology, which placed the descriptive precision of first-person experience at its core, it constitutes a ‘phenomenology mediated by third-person tools.’

However, as the ‘generative passages’ framework of Ramstead et al. (2022) argues, insofar as phenomenological description functions as a constraint on model building, the enterprise is justified as a methodological continuation of phenomenology. The experimental design of this paper fulfils precisely this condition: participants’ responses of ‘this resembles my experience’ or ‘this does not’ function as feedback constraining model parameters, and first-person description is retained as an indispensable component of the system. That is, this paper’s methodology does not abandon first-person description but rather transforms it into ‘operational positioning within stimulus space,’ thereby realising Varela’s ‘mutual constraints’ on a new technical medium. The extent to which this methodological turn preserves the essential insights of phenomenology (intentionality, embodiment, the irreducibility of lived experience) is a question that awaits further philosophical examination; in particular, integration with enactive embodiment remains a central outstanding challenge.

## 6 Conclusion

This paper has redefined computational phenomenology as a ‘generative passage,’ proposing a framework in which qualitative differences among altered states of consciousness are captured as variations in the manipulable descriptors of deep-learning objective functions and architectures. First-person differences are positioned not as mere reports but as formal requirements constraining model design, and are translated into operational variables that can be shuttled back and forth with third-person data.

To this end, the paper has foregrounded the objective functions and architectures of deep learning as a vocabulary for describing phenomenological differences. The effective causes exposed by DCNN visualisation operationally reformulate Huxley’s ‘reducing valve’ metaphor, routing it through the REBUS / Entropic Brain framework; the natural image prior of DGN/GANs computationally instantiates the constraint governing hallucinatory veridicality; and the GAN discriminator computationally instantiates prefrontal reality monitoring. Together, these afford a decomposition of hallucination mechanisms along three axes: effective-cause exposure (C), generative constraint (G), and reality-monitoring dysfunction (D). The reality-monitoring task based on iteration constitutes a methodological corollary that maps the internal dynamics of this generative–discriminative–classificatory framework onto behavioural measures, advancing computational phenomenology towards model identification and falsifiability.

At the same time, this framework is not a panacea. The biological plausibility of deep models, expansion to implementations incorporating neuromodulators and recurrent connectivity, and the extension of a vision-centric framework to multimodal phenomenology all remain open challenges. Nevertheless, the proposal advanced here, treating objective functions and architectures as ‘phenomenological descriptors’ and converting the generation-discrimination-classification framework into an experimentally tractable object, constitutes a step towards concretising, through a computational model, the mutually illuminating cycle in which phenomenology and neuroscience each refine the other. If consciousness is to be elevated from ‘a catalogue of correlations’ to ‘the machinery of generation,’ computational phenomenology has the potential to mature as a standard methodology cutting across the boundaries of theory, experiment, and clinical practice. Crucially, deep networks, while abstract models, draw architectural inspiration from neuroscience (feedforward visual hierarchy, adversarial interaction) and are supported by the evidence reviewed above. The framework of this paper is not a naive equation of GANs with the brain but rather a useful computational tool for generating testable insights. In this sense, computational phenomenology advances the ‘mutual illumination’ among phenomenology, computation, and neuroscience by one step, as a methodology that is both implemented and open to verification.

## Data Availability

The original contributions presented in the study are included in the article/supplementary material, further inquiries can be directed to the corresponding author.
